# Simultaneous Combination of the CDK4/6 Inhibitor Palbociclib With Regorafenib Induces Enhanced Anti-tumor Effects in Hepatocarcinoma Cell Lines

**DOI:** 10.3389/fonc.2020.563249

**Published:** 2020-09-23

**Authors:** Graziana Digiacomo, Claudia Fumarola, Silvia La Monica, Mara A. Bonelli, Daniele Cretella, Roberta Alfieri, Andrea Cavazzoni, Maricla Galetti, Patrizia Bertolini, Gabriele Missale, Pier Giorgio Petronini

**Affiliations:** ^1^Department of Medicine and Surgery, University of Parma, Parma, Italy; ^2^Istituto Nazionale per l'Assicurazione contro gli Infortuni sul Lavoro (INAIL) Research, Department of Occupational and Environmental Medicine, Epidemiology and Hygiene, Rome, Italy; ^3^Paediatric Hematology Oncology Unit, University Hospital of Parma, Parma, Italy; ^4^Unit of Infectious Diseases and Hepatology, University Hospital of Parma, Parma, Italy

**Keywords:** hepatocarcinoma (HCC), CDK4/6 inhibition, palbociclib (PD-0332991), regorafenib, metabolism

## Abstract

Advanced hepatocarcinoma (HCC) is an aggressive malignancy with poor prognosis and limited treatment options. Alterations of the cyclin D-CDK4/6-Rb pathway occur frequently in HCC, providing the rationale for its targeting at least in a molecular subset of HCC. In a panel of HCC cell lines, we investigated whether the CDK4/6 inhibitor palbociclib might improve the efficacy of regorafenib, a powerful multi-kinase inhibitor approved as second-line treatment for advanced HCC after sorafenib failure and currently under clinical investigation as first-line therapy in combination with immunotherapy. In Rb-proficient cells, the simultaneous drug combination, but not the sequential schedules, inhibited cell proliferation, either in short or in long-term experiments, and induced cell death more strongly than individual treatments. Moreover, the combination significantly reduced spheroid cell growth and inhibited cell migration/invasion. The superior efficacy of palbociclib plus regorafenib emerged also under hypoxia and was associated with a significant down-regulation of CDK4/6-Rb-myc and mTORC1/p70S6K signaling. Moreover, regorafenib suppressed palbociclib-induced expression of cyclin D1 contributing to the cytotoxic effects of the combination. Besides these inhibitory effects on cell viability/proliferation, palbociclib and regorafenib reduced glucose uptake, although this effect was dependent on the cell model and on the oxygen availability (normoxia or hypoxia). Palbociclib and regorafenib combination impaired glucose uptake and utilization, down-regulating basal and hypoxia-induced expression of HIF-1α, HIF-2α, GLUT-1, and MCT4 proteins as well as the activity/expression of glycolytic enzymes (HK2, PFKP, aldolase A, PKM2). In addition, regorafenib alone reduced mitochondrial respiration. The combined treatment impaired glucose metabolism and respiration without enhancing the effects of the single agents. Our findings provide pre-clinical evidence for the effectiveness of palbociclib and regorafenib combination in HCC cell models.

## Introduction

Hepatocellular carcinoma (HCC) is the most common primary liver malignancy, sixth most common cancer, and third leading cause of cancer deaths worldwide (1, 2). HCC is a highly heterogenic disease, typically diagnosed at late stages due to its prolonged asymptomatic nature. Only a small proportion of patients, diagnosed with early HCC, may benefit from potential curative therapies, such as liver transplantation, surgical resection, or local ablation (3). Therefore, systemic therapy remains the only available therapeutic option for advanced HCC. Sorafenib is a multi-kinase inhibitor with anti-proliferative and anti-angiogenic effects, targeting vascular endothelial growth factor receptor (VEGFR) 1–2–3, platelet derived growth factor receptor β (PDGFR-β), fms-like tyrosine kinase 3 (FLT-3), c-KIT, and RET. Sorafenib has been the only global first-line standard of care for advanced HCC until the recent Food and Drug Administration (FDA) first-line approval of the multi-kinase inhibitor lenvatinib (4). Regorafenib, an analog of sorafenib with improved target affinity and higher potency, was registered in 2017 as second-line treatment in patients who failed sorafenib therapy, based on the results from phase 3 RESORCE trial (5). More recently, other targeted drugs became available as second-line treatment options, i.e., the tyrosine kinase inhibitors (TKIs) cabozantinib and ramucirumab (6), and the immune checkpoint inhibitors nivolumab and pembrolizumab in the US (6–8). The combined use of atezolizumab, another immune checkpoint inhibitor (anti-PD-L1), and the anti-VEGF bevacizumab has been reported to be superior to sorafenib in a multicenter global phase III clinical trial (IMbrave150, NCT03434379) and FDA is considering this association as first-line treatment in HCC (9). Despite the recent advances in HCC therapy, precision medicine has not yet found clinical application and HCC cases are treated similarly, since the most common driver gene mutations in this disease (*TERT* promoter, *CTNNB1, TP53*, and *ARID1* mutations) have not yet been translated into effective therapeutic targets (10). Therefore, there is an urgent need for the identification of novel candidate targets for HCC therapy.

Previous studies have suggested that the retinoblastoma (Rb) pathway may play a role in this regard (11–13). This pathway controls the G1/S phase cell cycle transition through a mechanism involving cyclin dependent kinase 4/6 (CDK4/6)-cyclin D-mediated phosphorylation and inactivation of Rb protein, with consequent release of the transcription factor E2F. Besides this canonical function, novel roles have emerged for CDK4/6, cyclin D as well as E2F in other cellular processes, such as DNA damage repair, cell death, differentiation and metabolism, and immune modulation (14). Given the relevance of the Rb pathway for cancer progression, its pharmacological inhibition has gain attention as a promising treatment for a wide variety of cancers (15). Three CDK4/6 inhibitors, palbociclib (PD-0332991), ribociclib (LEE011) and abemaciclib (LY835219), are currently approved for patients with estrogen receptor (ER)-positive, human epidermal growth factor receptor 2 (HER2)-negative advanced or metastatic breast cancer in combination with an endocrine therapy (16). Aberrations of the components of the Rb pathway have been frequently reported in HCC: CDK4 overexpression was found in 73% of cases (17), *RB1* gene alterations and absence of p-Rb expression were detected in ~4–20% and 28% of cases, respectively (18, 19), whereas inactivation of the CDK inhibitor p16^INK4a^ emerged in 22% (18), 64% (19), or 58% (20) of patients. Based on these data and on the fact that the expression of a functional Rb protein is considered as a prerequisite for responsiveness to CDK4/6 inhibitors, at least a subset of HCC patients may benefit from treatment with these drugs.

Treatment with palbociclib has been previously proposed in combination with sorafenib, showing enhanced therapeutic effects in HCC tumor xenografts (12). Although sorafenib, and more recently lenvatinib, are the only approved front-line systemic treatment for HCC, there is an interest in evaluating the efficacy of regorafenib as first-line. Indeed, multiple lines of evidence indicate that regorafenib is more potent than sorafenib and a recent systematic comparison between the two drugs has shown that regorafenib is more effective in HCC mouse xenografts, significantly increasing their survival rate with less severe adverse reactions (21). Importantly, two clinical trials are ongoing to test the combination of regorafenib with the immune checkpoint inhibitors pembrolizumab (phase I study, HCCNCT03347292) (22) or tislelizumab (phase II study, NCT04183088) as first-line systemic therapy for patients with advanced HCC.

Based on these considerations, in this study we investigated whether the combination with palbociclib might represent a valuable alternative strategy to improve the anti-tumor activity of regorafenib in a panel of HCC cell lines. We demonstrated that this combination has superior efficacy compared with single agent treatments in both 2D and 3D cell systems, promoting enhanced cytotoxic effects under normoxic and hypoxic conditions, and impairing cell migration and invasion.

## Materials and Methods

### Cell Culture

Human HCC cell lines (HepG2, HUH7, PLC/PRF-5, HEP3B) were obtained from the American Type Culture Collection (ATCC, Manassas, VA); ATCC authenticates the phenotypes of these cell lines on a regular basis. The cells were cultured in Dulbecco's Modified Eagle Medium (DMEM) supplemented with 2 mM glutamine, 10% Fetal Bovine serum (FBS), and 100 U/ml penicillin/100 μg/ml streptomycin and incubated at 37°C in a humidified atmosphere of 5% CO_2_ in air. Hypoxic conditions (1% O_2_) were established by placing the cells in a tissue culture incubator with controlled O_2_ levels (Binder GmbH, Tuttlingen, Germany).

### Drug Treatments

Palbociclib (PD-0332991) and regorafenib (BAY73-4506) were purchased from Selleckchem (Houston, TX), and dissolved in water and DMSO, respectively. DMSO concentration never exceeded 0.1% (v/v); equal amounts of the solvent were added to control cells. Cobalt chloride (CoCl_2_) was purchased by Sigma-Aldrich (St. Louis, MO) and dissolved in water.

### Western Blotting

Western blot analysis was performed as previously described (23). Antibodies against p-Rb^Ser780^, Rb, cyclin D1, CDK4 and CDK6, *c*-Myc, p-AKT^Ser473^, AKT, p-mTOR^Ser2448^, mTOR, p-p70S6K^Thr389^, p70S6K, p-AMPKα1^Thr172^, p-ERK1/2^Thr202/Tyr204^, ERK1/2, p-PKM2^Tyr105^, PKM2 were from Cell Signaling Technology, Incorporated (Danvers, MA); anti-p-CDK6^Tyr24^ and anti-MCT4 were from Santa Cruz Biotechnology, Incorporated (Dallas, TX). Antibodies against CDKN2A/p16^INK4a^, AMPKα1, GLUT-1 were from Abcam (Cambridge, UK). Antibodies against HIF-1α and HIF-2α were from BD Biosciences (Franklin Lakes, NJ) and Novus Biologicals, LLC (Centennial, CO), respectively. Anti-β-actin (clone B11V08) was from BioVision (Milpitas, CA). Horseradish peroxidase-conjugated secondary antibodies and the chemiluminescence system were from Millipore (Millipore, MA). Reagents for electrophoresis and blotting analysis were from BIO-RAD Laboratories (Hercules, CA). For GLUT-1 detection, cells were lysed in GLUT-1 lysis buffer (1 % Triton X-100, 0.1% SDS, protease inhibitors) for 1 h on ice, and precleared by centrifugation for 10 min at 4°C. Protein extracts were denatured in sample buffer for 30 min before electrophoresis (24). The chemiluminescent signal was acquired by C-DiGit R Blot Scanner and the bands were quantified by Image Studio™Software, LI-COR Biotechnology (Lincoln, NE).

### Analysis of Cell Proliferation and Cell Death

Cell viability/proliferation was evaluated by counting the cells in a Bürker hemocytometer with trypan blue exclusion method and by Crystal Violet (CV) staining. Cell death was analyzed by fluorescence microscopy after staining with Hoechst 33342 and Propidium Iodide (PI), as described elsewhere (25). The nature of the interaction between palbociclib and regorafenib was calculated using the Bliss additivism model (25). A theoretical dose–response curve was calculated for combined inhibition using the equation E_bliss_ = EA + EB – EA × EB, where EA and EB are the percent of inhibition vs. control cells, obtained by regorafenib (A) and palbociclib (B) alone and E_bliss_ is the percent of inhibition that would be expected if the combination was exactly additive. If the combination effect is higher than the expected Bliss equation value, the interaction is synergistic, while if the effect is lower, the interaction is antagonistic.

### Quantitative Real-Time PCR

Total RNA was isolated by RNeasy Mini Kit (Qiagen, Venlo, Netherlands) and the quantitative real-time polymerase chain reaction (PCR) was performed using the StepOne system instrument (Applied Biosystems) as previously described (23). The primers for GLUT-1 (QT00068957), HK2 (QT00013209), PFKP (QT00007147), and aldolase A (QT00082460) were purchased from Qiagen. The fold change was calculated by the ΔΔCT method.

### Spheroid Generation and Growth

Spheroids from HCC cells were generated using LIPIDURE^®^-COAT PLATE A-U96 (NOF Corporation, Japan) according to manufacturer's instruction. Briefly, 3 days after seeding the spheroids were formed and treated with drugs or vehicle for 4 or 8 days. The effects of the drugs were evaluated in terms of volume changes using the Nikon Eclipse E400 Microscope with digital Net camera. The volume of spheroids [*D* = (*D*max + *D*min)/2; *V* = 4/3π (*D*/2)3] was measured using SpheroidSizer, a MATLAB-based and open-source software application (26).

### Migration and Invasion

The migration and invasion assays were carried out using Transwell chambers with 6.5-mm diameter polycarbonate filters (8μm pore size, BD Biosciences, Erembodegem, Belgium), uncoated or coated with Matrigel™, and were performed as previously described (27).

### Metabolic Assays

#### Uptake

Glucose uptake was assessed using the nonmetabolizable analog Deoxy-D-glucose-2-[1,2-3H(N)] (2DG, PerkinElmer, Waltham, MA), as described previously (24).

#### OCR and ECAR Measurements

The oxygen consumption rate (OCR) and extracellular acidification rate (ECAR) were measured by a Seahorse Extracellular Flux XFp analyzer (Seahorse Bioscience, North Billerica, MA, USA) according to the manufacturer's instructions.

Mitochondrial respiration was analyzed using the Seahorse XF Cell Mito Stress Test kit. Baseline respiration levels were measured by subtracting non-mitochondrial respiration (OCR values obtained after injection of antimycin A/Rotenone) from initial OCR levels. The OCR drop observed after the injection of the ATP synthase inhibitor oligomycin reflected the ATP-linked respiration. The following injection of FCCP collapsed the mitochondrial membrane potential and brought OCR to its maximum. Finally, the injection of antimycin A and Rotenone, inhibitors of complex III and I respectively, blocked the mitochondrial respiratory chain and strongly inhibited the respiration. The spare respiratory capacity was calculated as the difference between the maximal and the basal OCR values and measured the amount of extra ATP that could be produced by oxidative phosphorylation (OXPHOS) in the case of a rapid increase in energy demand or under a metabolic stress condition.

The OCR and ECAR were expressed in pmol/min and mpH/min, respectively, and normalized to the μg of proteins of each sample.

### Statistical Analysis

Statistical analyses were carried out using Graph- Pad Prism version 6.0 software (GraphPad Software, San Diego, CA). Statistical significance of differences among data was estimated by Student's *t*-test or by analysis of variance (ANOVA) followed by Tukey's post-test, and *p*-values are indicated where appropriate in the figures and in their legends. *p* < 0.05 were considered significant.

## Results

### A Functional Rb Pathway Is a Predictive Factor of Response to Palbociclib in HCC Cell Lines

**Figure 1:**
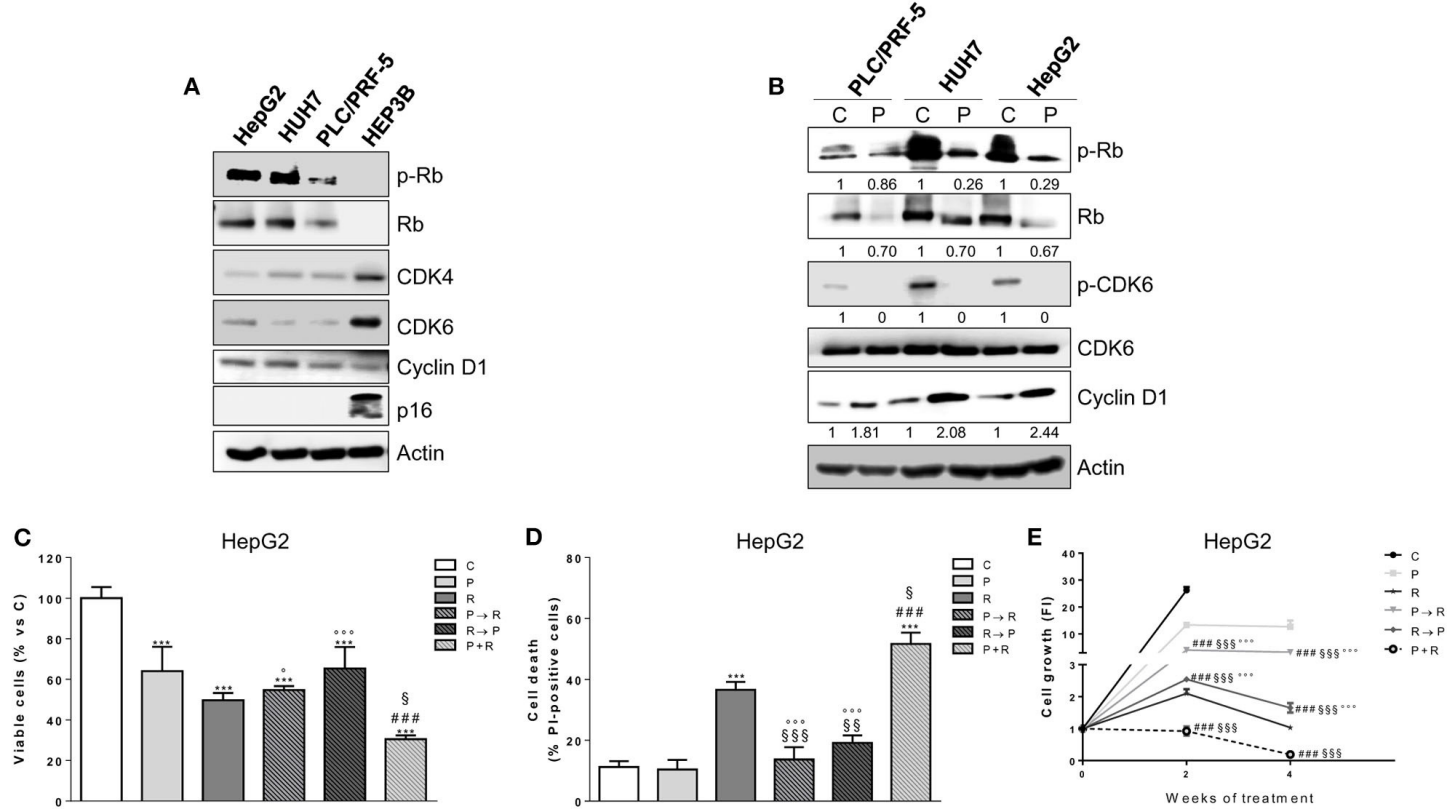
Palbociclib alone inhibits the proliferation of Rb-positive HCC cell lines and in simultaneous combination with regorafenib enhances its cytotoxicity. **(A)** HCC cells (HepG2, HUH7, PLC/PRF-5, and HEP3B) were analyzed by Western blotting for the basal expression of cell cycle regulatory proteins. **(B)** PLC/PRF-5, HUH7, and HepG2 cell were untreated (Control, C) or treated with 1 μM palbociclib (P) for 24 h. The cells were lysed and the expression of the indicated proteins was evaluated by Western blotting. HepG2 cells were treated with 0.1 μM P or 1 μM regorafenib (R) for 48 h or with different combinations of the two drugs: P for 24 h followed by R for 24h (P → R), R for 24 h followed by P for 24 h (R → P), or a simultaneous combination for 48 h (P+R). The cells were counted using the trypan blue dye exclusion method **(C)** and the percentage of cell death was evaluated after Hoechst 33342 and PI staining **(D)**. Data are expressed as percent vs. control or as percent of dead cells, respectively. ^***^p < 0.001 vs. C; ^###^*p* < 0.001 vs. P; ^§^*p* < 0.05, ^§§^*p* < 0.01, ^§§§^*p* < 0.001 vs. R; °*p* < 0.05, °°°*p* < 0.001 vs. P+R. **(E)** HepG2 cells were treated with the different schedules reported in panels **(C,D)** for 2 or 4 weeks. The growth medium with drugs was refreshed every 3 days. Cell proliferation was evaluated by CV assay. Data are expressed as Fold Increase (FI). The FI index was calculated as the ratio between cell proliferation after 2 or 4 weeks and cell proliferation at T_0_ (T_0_ = 24 h after seeding). ^###^*p* < 0.001 vs. P; ^§§§^*p* < 0.001, vs. R; °°°*p* < 0.001 vs. P+R. All the data reported are representative of two independent experiments.

We evaluated the effects of palbociclib on cell proliferation in a panel of HCC cell lines. HepG2, HUH7, and PLC/PRF-5 cells were sensitive to the drug, with IC_50_ values of 0.1 ± 0.03, 0.1 ± 0.05, and 0.3 ± 0.12 μM at 6 days, respectively; in contrast, HEP3B cells showed IC_50_ values > 3 μM, and were therefore considered resistant. Accordingly, only palbociclib-sensitive cell models expressed p-Rb and Rb protein (Figure 1A), confirming previous suggestions that the expression of a functional Rb protein may serve as a predictive factor of response to CDK4/6 inhibitors (28). CDK4 and CDK6 protein expression levels were higher in the resistant than in the sensitive cells. In addition, the sensitive cell models expressed higher levels of cyclin D1 protein as compared with HEP3B cells, but were negative for the expression of the cell cycle inhibitor p16^INK4a^, which in contrast was expressed in the resistant cells (Figure 1A). These results are in line with previous pre-clinical studies showing that cyclin D1 expression and the loss of p16^INK4a^ correlate with a superior efficacy of CDK4/6 inhibitors in breast cancer cells (29–31). Exposure of sensitive HCC cells to palbociclib decreased CDK6 phosphorylation because of the inhibition of cyclin D1-CDK4/6 complexes (Figure 1B). As a result, Rb was hypo-phosphorylated, and total Rb protein levels were down-regulated, whereas cyclin D1 expression increased in all three cell models analyzed (32, 33).

### The Simultaneous Combination of Palbociclib With Regorafenib Inhibits Cell Proliferation and Induces Cell Death More Efficaciously Than Sequential Treatments

We sought to investigate whether palbociclib treatment could improve the efficacy of the multi-kinase inhibitor regorafenib. Treatment with regorafenib alone inhibited cell proliferation in a dose-dependent manner in all palbociclib-sensitive cell models, although it was more effective in HepG2 and HUH7 cells (IC_50_ values of 1.1 ± 0.08 and 1.6 ± 0.14 μM at 3 days, respectively) than in PLC/PRF-5 cells (IC_50_ value of 7 ± 0.92 μM), confirming results from previous studies (34, 35). Given that combinations of palbociclib with other agents have been proposed in sequential regimens (23, 25, 36), we investigated the effects of combining palbociclib with regorafenib on cell proliferation and cell death comparing a simultaneous treatment with sequential schedules. In HepG2 cells the simultaneous combination not only caused a greater inhibition of cell proliferation, but also promoted a stronger induction of cell death compared with single agent treatments, as shown in Figures 1C,D. In contrast, these enhanced effects were lost when the drugs were combined sequentially (palbociclib treatment followed by regorafenib or regorafenib followed by palbociclib). The effects of these different schedules were also assessed in long-term experiments in HepG2 cells (Figure 1E). Again, the simultaneous treatment exerted a stronger anti-tumor activity in comparison with individual treatments, while both the sequential treatments were even less effective than regorafenib alone. It is worth noting that palbociclib potentiated also the growth-inhibitory effects of sorafenib in HepG2 cells (Supplementary Figure 1), as previously shown in HCC *in vivo* models (12); however, the combination with regorafenib demonstrated a superior efficacy, being regorafenib more effective. Altogether, these results prompted us to choose the simultaneous combined treatment with palbociclib and regorafenib for the subsequent experiments.

### The Simultaneous Combination of Palbociclib With Regorafenib Inhibits Cell Growth of Spheroids and Reduces Cell Migration and Invasion

**Figure 2:**
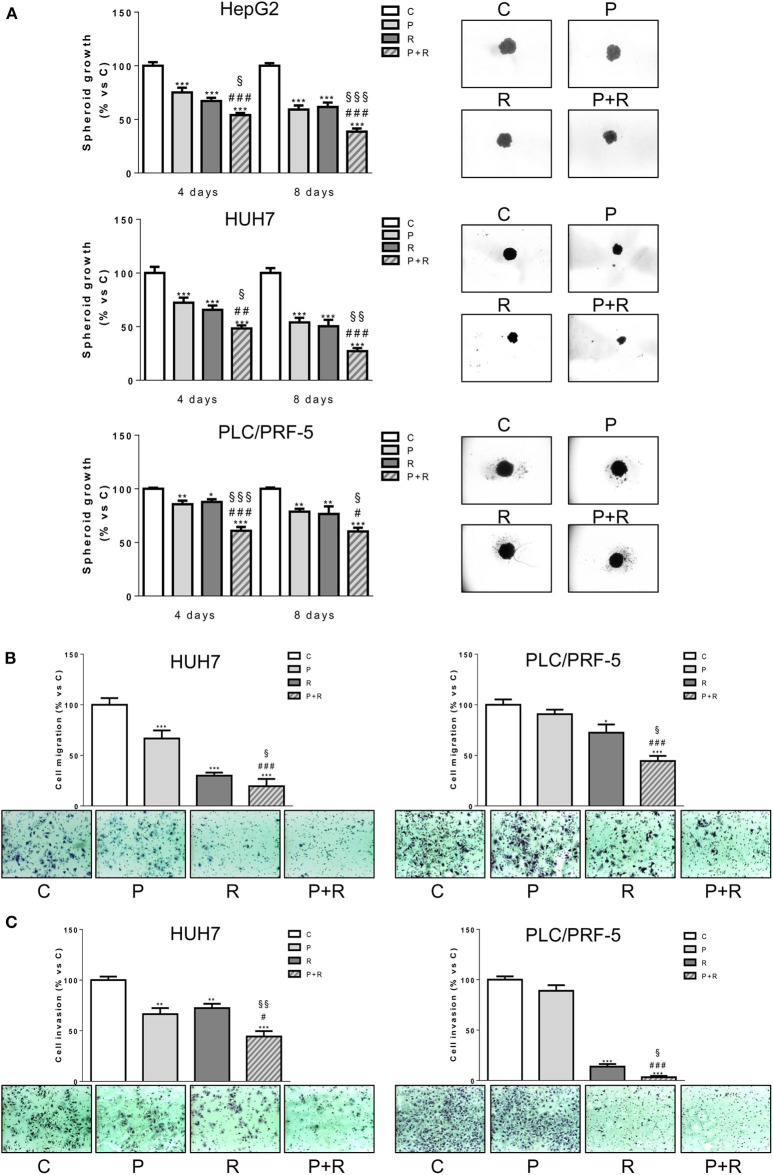
Palbociclib combined with regorafenib inhibits 3D cell growth and reduces cell migration and invasion. **(A)** The growth of spheroids from HepG2, HUH7, and PLC/PRF-5 was analyzed after 4 or 8 days of treatment with 0.1 μM P for HepG2 and HUH7, 0.5 μM P for PLC/PRF-5, and 1, 1.5, or 7 μM R for HepG2, HUH7, and PLC/PRF-5, respectively, as single treatment or in combination. The data are expressed as percent of spheroid growth vs. control. Representative images of spheroids after 8 days of culture are shown. ^*^p < 0.05, ^**^p < 0.01, ^***^p < 0.001 vs. C, ^#^*p* < 0.05, ^##^*p* < 0.01, ^###^*p* < 0.001 vs. P; ^§^*p* < 0.05, ^§§^*p* < 0.01, ^§§§^*p* < 0.001 vs. R. **(B,C)** HUH7 and PLC/PRF-5 cells were treated with 1 μM P or 1.5 μM R for HUH7 and 7 μM R for PLC/PRF-5, as single treatment or in combination for 18 h. Migrated or invaded cells were then counted. Data are expressed as percent vs. control. Representative fields of migration or invasion are shown (magnification of 100×). ^*^p < 0.05, ^**^p < 0.01, ^***^p < 0.001 vs. C, ^#^*p* < 0.05, ^###^*p* < 0.001 vs. P; ^§^*p* < 0.05, ^§§^*p* < 0.01 vs. R. Data in panel **(A)** are mean values ±SD of three independent experiments. Data in panels **(B,C)** are representative of two independent experiments.

We investigated the effect of palbociclib combined with regorafenib on cell growth of 3D tumor spheroids (Figure 2A). Both palbociclib and regorafenib were capable of inhibiting spheroid cell growth and, most importantly, the combined treatment was even more effective in all three HCC cell models. In addition, we used HUH7 and PLC/PRF-5 cells to investigate the effect of the drug combination on migration and invasion (Figures 2B,C). The single agents reduced the number of migrating and invading HUH7 cells, whereas in PLC/PRF-5 cells only regorafenib was effective. However, the drug combination decreased cell migration and invasion more strongly than single treatments in both cell models.

### The Simultaneous Combination of Palbociclib With Regorafenib Is More Effective Than Single Treatments Also Under Hypoxia

**Figure 3:**
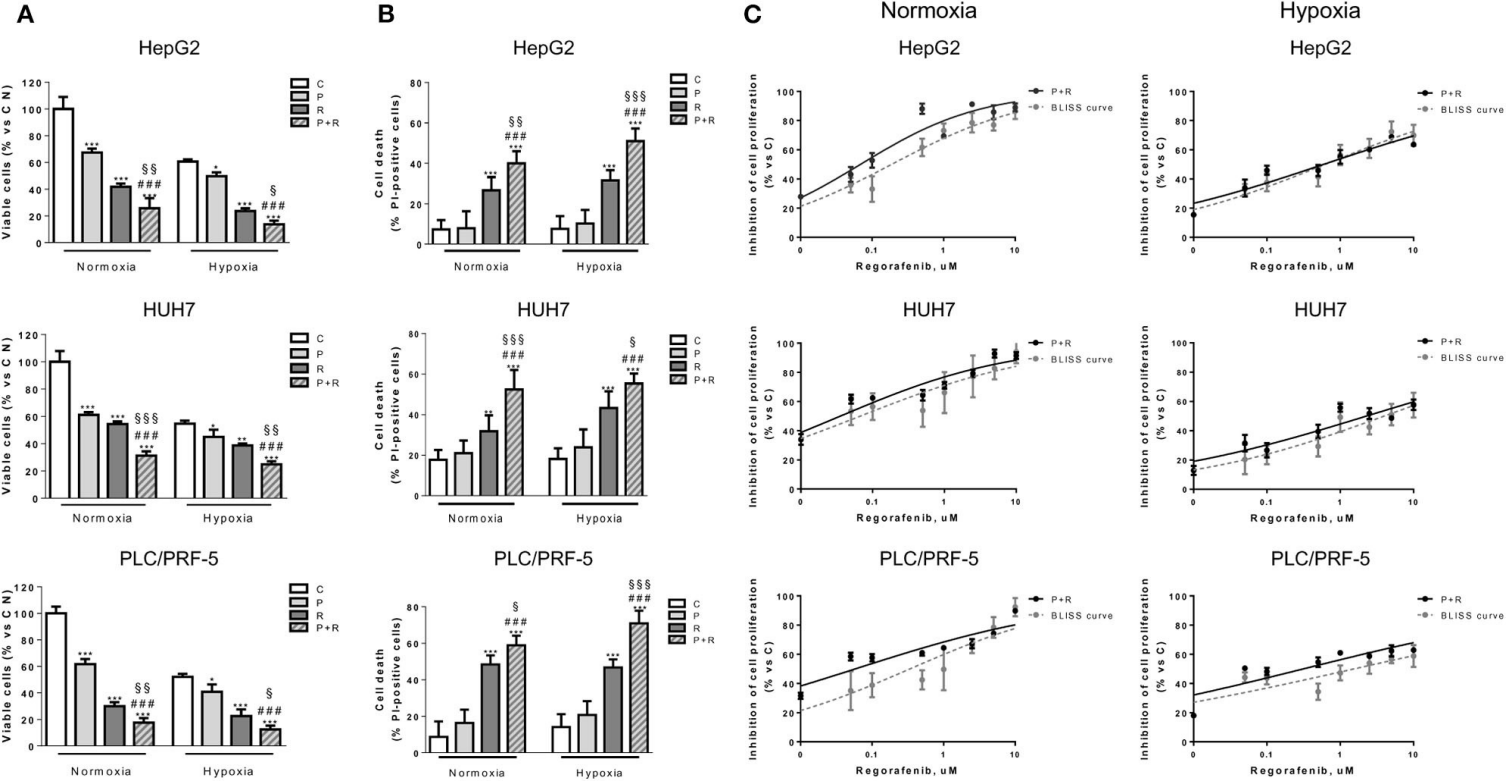
Palbociclib combined with regorafenib inhibits cell proliferation/viability with additive effects and induces cell death under both normoxic and hypoxic conditions. HepG2, HUH7 were incubated with 0.1 μM P, 1 or 1.5 μM R, respectively, PLC/PRF-5 with 0.5 μM P, 7 μM R, alone or in simultaneous combination under normoxic or hypoxic conditions. After 72 h, the cells were counted using the trypan blue dye exclusion method **(A)** and the percentage of cell death was evaluated after Hoechst 33342 and PI staining **(B)**. Data are expressed as percent vs. control cells in normoxia or as percent of dead cells, respectively. ^*^p < 0.05, ^**^p < 0.01, ^***^p < 0.001 vs. C; ^###^*p* < 0.001 vs. P; ^§^*p* < 0.05, ^§§^*p* < 0.01, ^§§§^*p* < 0.001 vs. R. **(C)** HepG2, HUH7 were treated with 0.1 μM P and PLC/PRF-5 with 0.5 μM P in combination with increasing concentrations of R (from 0.05 nM to 10 μM) under normoxic or hypoxic conditions. After 72 h, cell proliferation was assessed by CV assay. The type of interaction (antagonistic, additive, or synergistic) was evaluated through Bliss analysis. Data are expressed as percent inhibition vs. control. Data in panels **(A,B)** are representative of two independent experiments. Data in panel **(C)** are representative of three independent experiments.

Considering that treatments with anti-angiogenic drugs like regorafenib may exacerbate the hypoxic conditions of the tumor microenvironment, we evaluated the effects of palbociclib plus regorafenib also under hypoxia. As shown in Figures 3A,B, the drug combination reduced the number of viable cells more effectively than individual treatments, significantly increasing the percentage of dead cells, not only under normoxic but also under hypoxic conditions in HepG2, HUH7, and PLC/PRF-5 cells. It is worth noting that hypoxia *per se* reduced cell proliferation, without affecting cell death, and that the drug inhibitory activity on cell proliferation was less evident under this condition.

To establish the nature of the interaction between palbociclib and regorafenib, we tested the efficacy of combining a fixed dose of palbociclib with increasing concentrations of regorafenib and demonstrated, using the Bliss experimental model, that such combination produced additive inhibitory effects on cell proliferation in all cell lines analyzed under either normoxia or hypoxia (Figure 3C).

**Figure 4:**
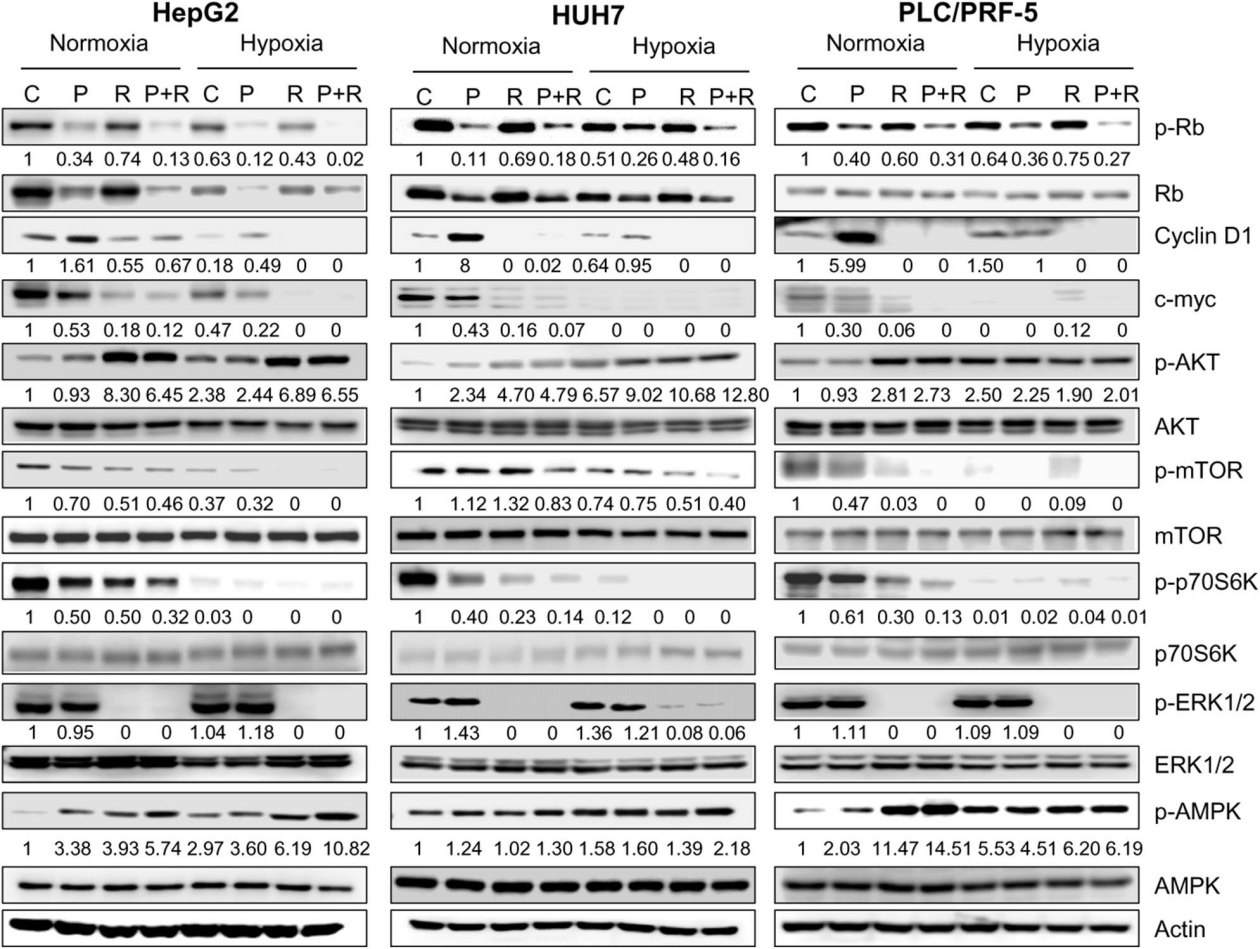
Effects of palbociclib and regorafenib simultaneous combination on the activation/expression of cell cycle regulatory proteins and intracellular signaling pathways. HepG2, HUH7, and PLC/PRF-5 cells were treated with 1 μM P or 1, 1.5, or 7 μM R, respectively, as single treatment or in combination under normoxic and hypoxic conditions for 24 h. The cells were lysed and the expression of the indicated proteins was evaluated by Western blot analysis. The results are representative of two independent experiments.

To get insights into the molecular mechanisms underlying the superior efficacy of palbociclib/regorafenib combination, we analyzed its effects on the activation/expression of cell cycle regulatory proteins along with components of the AKT and MAPK signaling pathways in all three HCC cell models. As shown in Figure 4, the levels of p-Rb and total Rb protein were down-regulated by palbociclib treatment and further decreased after combination with regorafenib under both normoxic and hypoxic conditions. On the other hand, cyclin D1 was up-regulated by palbociclib, and regorafenib reduced or even completely inhibited both the basal level and palbociclib-induced expression of this protein. In addition, the transcription factor *c*-myc, whose interplay with E2F is well-recognized (37–39), was down-regulated by palbociclib and more strongly by regorafenib alone or in combination. Interestingly, the mammalian target of rapamycin C1 (mTORC1) signaling was down-regulated by both drugs and further inhibited by the combined treatment, as demonstrated by the decreased phosphorylation of the mTORC1 downstream target p70S6K. Of note, hypoxia *per se* significantly reduced both *c*-myc and p-p70S6K levels, thus rendering the drug effects less appreciable. Palbociclib and regorafenib-mediated-inhibition of p70S6K phosphorylation was not downstream of AKT, which canonically modulates the mTORC1/p70S6K signaling; indeed, both drugs alone or in combination activated instead of inhibiting AKT. Conversely, the mitogen activated protein kinase (MAPK) pathway, which can modulate the mTORC1/p70S6K signaling independently of AKT (40), was not affected by palbociclib, as indicated by the maintenance of ERK1/2 phosphorylation, while it was completely inhibited by regorafenib alone or in combination. Therefore, inhibition of the MAPK pathway might contribute to p70S6K down-regulation (41) mediated by regorafenib but not by palbociclib. Interestingly, both drugs, and even more the combination, activated AMPK, a master energy sensor that plays a key role in the adaptive responses to stressful conditions, and that might be involved in the observed inhibition of the mTORC1 signaling.

### Effects of the Simultaneous Combination of Palbociclib With Regorafenib on Energy Metabolism

**Figure 5:**
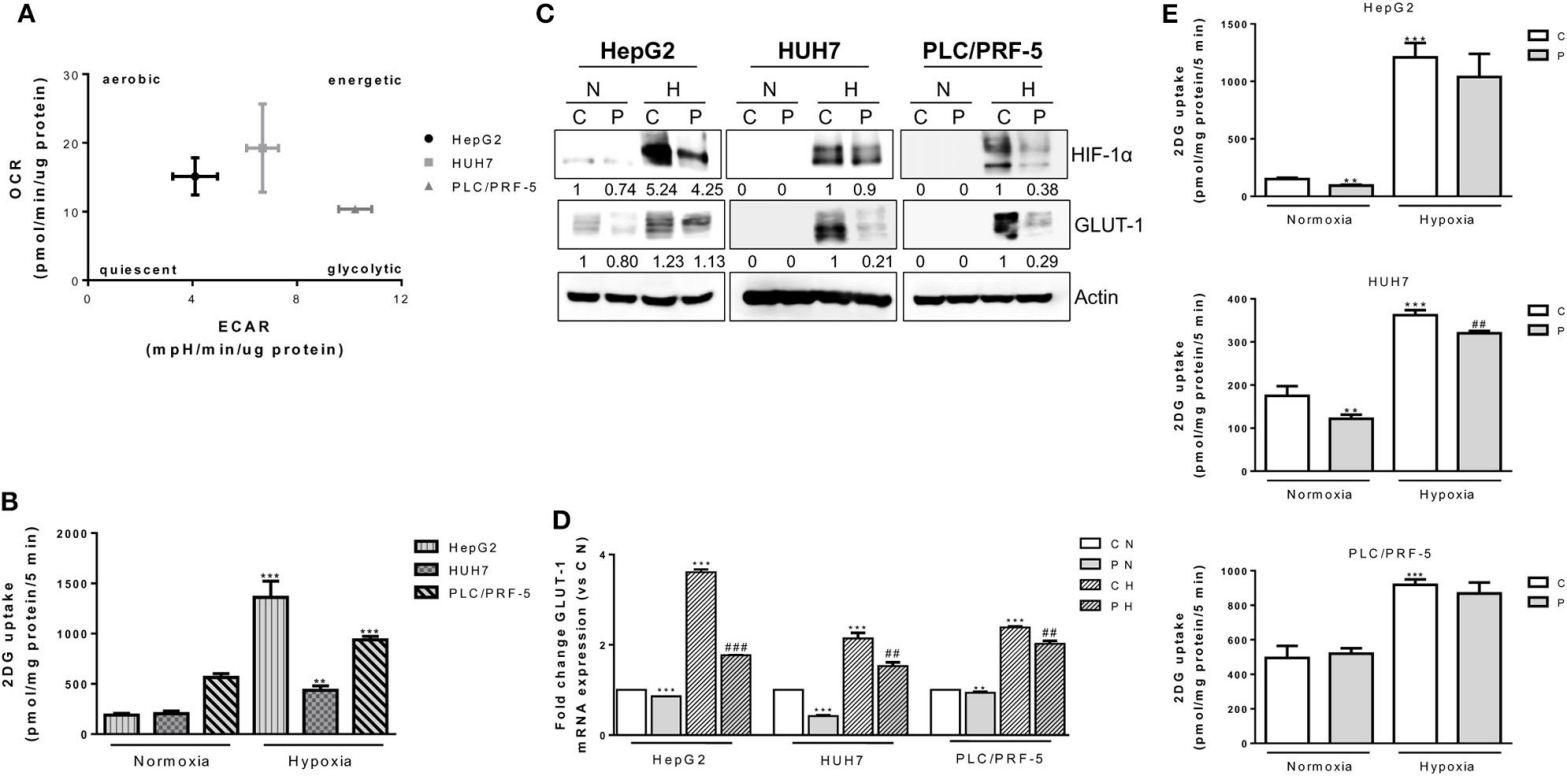
Effects of palbociclib alone on glucose metabolism. **(A)** HepG2, HUH7, and PLC/PRF-5 cells were analyzed for OCR and ECAR in basal conditions. Data are expressed as pmol/min/μg protein and mpH/min/μg protein, respectively. **(B)** HepG2, HUH7, and PLC/PRF-5 cells were exposed to hypoxia for 24 h and then analyzed for glucose uptake. Data were calculated and expressed as pmol of 2DG/mg protein/5 min. ^**^p < 0.01, ^***^p < 0.001 vs. the corresponding value in normoxia. **(C)** HepG2, HUH7, and PLC/PRF-5 cells were treated with 1 μM P under normoxic (N) and hypoxic (H) conditions for 24 h. The cells were lysed and the expression of the indicated proteins was evaluated by Western blot analysis. **(D)** The cells were treated as in panel **(C)** and, after cell lysis, GLUT-1 mRNA expression levels were analyzed by RT-PCR. Results are plotted as 2^−ΔΔCT^ ± SD. ^**^p < 0.01, ^***^p < 0.001 vs. C N; ^##^*p* < 0.01, ^###^*p* < 0.001 vs. C H. **(E)** HepG2, HUH7, and PLC/PRF-5 cells were treated with 1 μM P under normoxic and hypoxic conditions for 24 h and then glucose uptake was measured. Data were calculated and expressed as pmol of 2DG/mg protein/5 min. ^**^p < 0.01, ^***^p < 0.001 vs. C in normoxia; ^##^*p* < 0.01 vs. C in Hypoxia. All data are representative of two independent experiments.

It is now recognized that cell cycle-proteins are involved in the regulation of cell energy metabolism (42). Therefore, we investigated whether a contribution to the anti-proliferative action of palbociclib may also derive from metabolic effects, an aspect that has not yet been elucidated in HCC models. Firstly, we analyzed the metabolic status of the HCC cell models, and determined that PLC/PRF-5 cells were the most glycolytic and less dependent on mitochondrial respiration (Figure 5A). Accordingly, these cells showed a higher glucose uptake as compared with the other cell models (Figure 5B). However, when the cells were incubated under hypoxic conditions, the less glycolytic HepG2 cells showed the highest induction of glucose uptake, indicating a high metabolic plasticity (Figure 5B). As shown in Figure 5C, palbociclib significantly reduced the expression of both hypoxia-inducible factor 1 α (HIF-1α) and GLUT-1 glucose transporter in all three models; GLUT-1 was also down-regulated at mRNA level, indicating a transcriptional modulation presumably consequence of HIF-1α inhibition (Figure 5D). Surprisingly, these effects did not result in the inhibition of glucose uptake in all HCC cell models (Figure 5E). Indeed, both HepG2 and HUH7 cells treated with palbociclib showed a reduced glucose uptake under normoxia, but this effect was observed under hypoxia only in HUH7 cells. In contrast, palbociclib treatment did not affect glucose uptake in PLC5/PRF-5 cells under either normoxia or hypoxia. These results suggest that glucose uptake may be differently modulated in HCC cells, possibly involving glucose transporters other than GLUT-1 as well as HIF-1α-independent mechanisms that are not affected by palbociclib.

**Figure 6:**
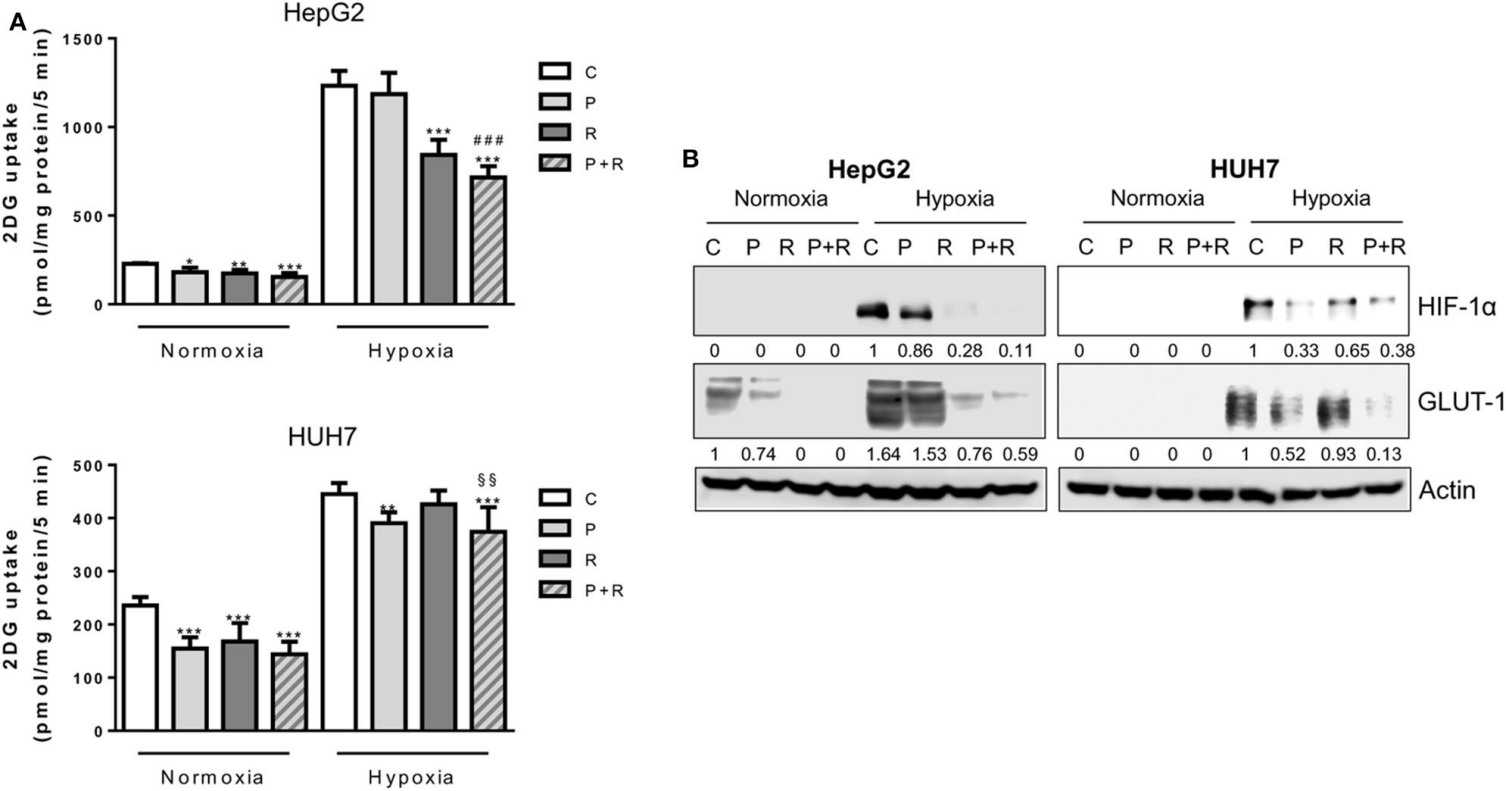
Effects of palbociclib and regorafenib combination on energy metabolism. **(A)** HepG2 and HUH7 cells were treated with 1 μM P or 1 and 1.5 μM R, respectively, alone or in combination, under normoxic and hypoxic conditions for 24 h. Glucose uptake was then measured. Data were calculated and expressed as pmol of 2DG/mg protein/5 min and are mean values ±SD of three independent experiments. ^*^p < 0.05, ^**^p < 0.01, ^***^p < 0.001 vs. C; ^###^*p* < 0.001 vs. P; ^§§^*p* < 0.01 vs. R. **(B)** HepG2 and HUH7 cells, treated as in panel **(A)**, were lysed and the expression of the indicated proteins was evaluated by Western blot analysis. Results are representative of two independent experiments.

Then, we evaluated whether palbociclib combined with regorafenib might affect glucose metabolism. Regorafenib also was effective in reducing the glucose uptake under normoxic conditions, confirming previous findings (43); however, the drug combination failed to potentiate the inhibitory effects of single treatments (Figure 6A). Under hypoxia, the strong inhibition of glucose uptake observed in HepG2 cells treated with the combination was mediated only by regorafenib (Figure 6A). In addition, HIF-1α and GLUT-1 expression were almost completely inhibited by regorafenib alone and in combination (Figure 6B). In contrast, in HUH7 cells palbociclib and not regorafenib was mainly responsible for the down-regulation of HIF-1α/GLUT-1 expression and glucose uptake associated with the combined treatment (Figures 6A,B).

Then, we further investigated the effects of palbociclib/regorafenib combination on glucose metabolism in HepG2 cells.

**Figure 7:**
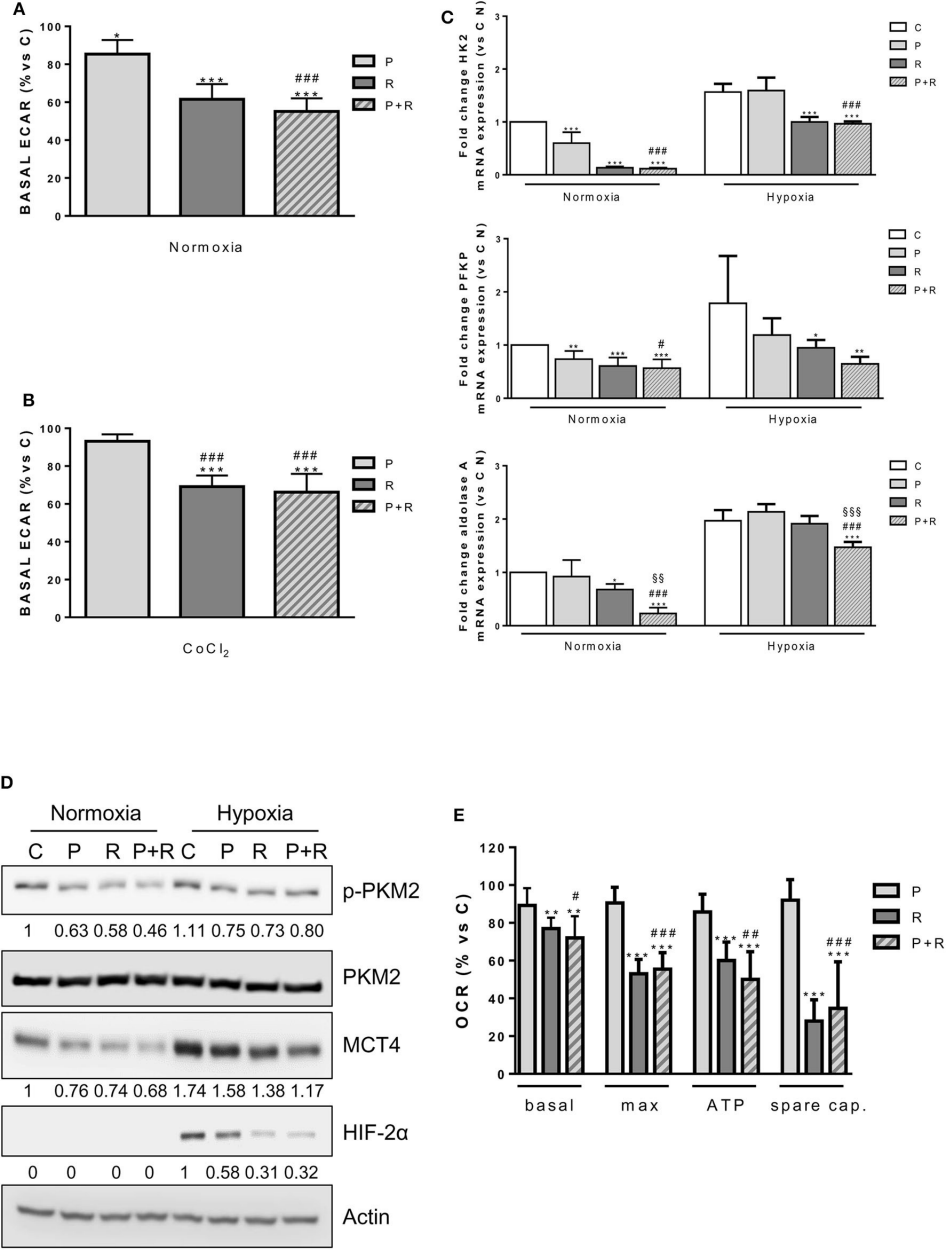
**(A)** HepG2 cells were treated with 1 μM P or 1 μM R alone or in combination for 24 h. Then, basal ECAR was measured by seahorse. **(B)** HepG2 cells were treated with 1 μM P or 1 μM R alone or in combination in the presence of 200 μM CoCl_2_ for 24 h. Then, basal ECAR was measured by seahorse. **(C)** HepG2 cells were treated as in panel **(A)** under normoxic and hypoxic conditions for 24 h. After cell lysis, HK2, PFKP, and aldolase A mRNA expression levels were analyzed by RT-PCR. Results are plotted as 2^−ΔΔCT^ ± SD. **(D)** HepG2 cells were treated as in panel **(A)** under normoxic and hypoxic conditions, were lysed and the expression of the indicated proteins was evaluated by Western blot analysis. **(E)** HepG2 cells were treated as in panel **(A)** and analyzed for the mitochondrial function through the seahorse mitochondrial stress test. Data are mean values ±SD of two **(E)** or three **(A–C)** independent experiments. ^*^p < 0.05, ^**^p < 0.01, ^***^p < 0.001 vs. C; ^#^*p* < 0.05, ^##^*p* < 0.01, ^##^*p* < 0.001 vs. P; ^§§^*p* < 0.01, ^§§§^*p* < 0.001 vs. R. Results in panel **(D)** are representative of three independent experiments.

Firstly, we demonstrated that the ECAR, an indicator of anaerobic glycolysis based on the production of lactic acid, was decreased by palbociclib and regorafenib treatment under normoxia, although no further reduction was promoted by the combination, thus reflecting the effects on glucose uptake (Figure 7A). In addition, when HepG2 cells were treated with CoCl_2_, which mimics exposure to hypoxic conditions, only regorafenib and the combination significantly reduced the ECAR (Figure 7B).

Under normoxic conditions, both palbociclib and regorafenib as well as their combination down-regulated the expression of hexokinase 2 (HK2), the major HK isoform expressed in HCC (44), and phosphofructokinase platelet (PFKP), a major isoform of PFK-1 controlling the first rate-limiting step of glycolysis in cancer cells (45) (Figure 7C). Under hypoxic conditions, both genes were significantly down-regulated by regorafenib and no further decrease was induced by the combination. In contrast, the expression of aldolase A, which is up-regulated in HCC tissues and correlates with HCC malignancy (46), was significantly decreased by the combination in comparison with control or single agent treatments (Figure 7C). Then, we investigated the activation/expression of pyruvate kinase M2 (PKM2), which is predominantly expressed as a dimeric form with low PK activity in cancer cells (47). As shown in Figure 7D, palbociclib and regorafenib alone or in combination had no effect on PKM2 protein expression, but decreased its phosphorylation at Tyr^105^, presumably favoring the switch from the low active dimer to the highly active tetrameric form of the enzyme (48).

In addition, the single agents and their combination reduced the expression of monocarboxylate transporter 4 (MCT4), which is responsible for the efflux of cytosolic lactate in multiple cancers including HCC (49). Interestingly, palbociclib and more strongly regorafenib alone and in combination down-regulated HIF-2α under hypoxia (Figure 7D), thereby impeding the compensatory mechanism that increases the expression of this isoform when HIF-1α is inhibited (50).

A variety of evidence indicates that regorafenib affects mitochondrial function by acting as an uncoupler of OXPHOS, promoting cytochrome c release, and decreasing the mitochondrial membrane potential (51, 52). Therefore, we analyzed the impact of palbociclib, regorafenib, and their combination on mitochondrial function in HepG2 cells by measuring the OCR in basal conditions and upon exposure to the mitochondrial stressors oligomycin and FCCP. As shown in Figure 7E, only regorafenib significantly reduced the basal and ATP-linked respiration as well as the maximal and spare respiratory capacities, and the combination did not produce any further inhibitory effect.

## Discussion

In this pre-clinical study, we demonstrate that the simultaneous combination of the CDK4/6 inhibitor palbociclib with regorafenib induces enhanced anti-tumor effects in HCC cell models.

Alterations of cell cycle regulators, such as increased CDK4 expression/activity or p16^INK4a^ inactivation, have been frequently reported in HCC, providing the rationale to test CDK4/6 inhibitors for HCC treatment (53). A multicenter single arm phase II trial is ongoing to evaluate the efficacy of palbociclib as second-line therapy after sorafenib failure (NCT01356628), supported by pre-clinical data demonstrating palbociclib anti-tumor activity in HCC cell lines as well as in xenograft models (12, 54, 55). Here we confirm that palbociclib was effective in inhibiting cell growth only in Rb-expressing, p16^INK4a^ negative HCC cells, supporting the role of Rb in predicting the HCC response to CDK4/6 inhibition. Indeed, despite HCC cells subjected to acute Rb knockdown were shown to retain short-term sensitivity to palbociclib, due to compensatory mechanisms involving the p107 Rb family member (54), Rb loss has proven to be responsible for the acquisition of palbociclib resistance (12).

In these Rb-proficient HCC cells, palbociclib exerted a cytostatic effect. In other studies, palbociclib was shown to induce apoptosis in some cancer cell models (56) and also in HCC cells independently of Rb expression (55), although this effect might depend on the high concentrations used. Actually, the potential of palbociclib to induce cancer cell death in monotherapy is generally limited (57). However, pre-clinical data on multiple cancer models indicate that the predominantly reversible cytostatic responses to palbociclib or other CDK4/6 inhibitors can be converted into irreversible cell cycle arrest and senescence or apoptosis through combinations with other targeted drugs (23, 25, 58, 59) or chemotherapy (60). In HCC mouse models, palbociclib has been successfully associated with sorafenib, with enhanced reduction of tumor growth or even tumor regression (12). Here, we demonstrate that palbociclib treatment in HCC cells provides superior efficacy when combined with regorafenib, leading to additive anti-proliferative effects and enhancing regorafenib-mediated cytotoxicity. In particular, these results were achieved when the two drugs were simultaneously combined, either in short- or long-term experiments, while the sequential schedules (palbociclib before or after regorafenib) failed to improve the anti-tumor response compared with single agent treatments. These results may be explained considering that palbociclib usually induces a reversible cell cycle arrest in HCC cells and treatment discontinuation may result in tumor regrowth (12), a relevant aspect that should be taken into account when planning combined therapies with palbociclib.

The effectiveness of palbociclib plus regorafenib combination observed in HCC cell models was associated with a significant down-regulation of CDK4/6-Rb-myc and mTORC1/p70S6K signaling. It is now recognized the existence of a cross-talk between the CDK4/6-Rb pathway and the mTOR pathway. CDK4/6 inhibition has been shown to reduce mTORC1 activity in some cancer models (61, 62), although in some others the opposite effect has been described (23, 25, 63), indicating the existence of cell-type specific regulatory mechanisms. On the other hand, mTOR inhibitors down-regulate Rb protein, blocking the G1 to S phase transition of the cell cycle. This inhibitory effect has been ascribed to the suppression of p70S6K phosphorylation/activation (40). Besides mTORC1, another upstream regulator of p70S6K is ERK1/2, which can directly phosphorylate and activate p70S6K, leading to the activation of the transcription factor CREB (64). Therefore, in our experimental system, palbociclib-mediated inhibition of the CDK4/6-Rb axis together with regorafenib-dependent suppression of ERK1/2 activity resulted in a stronger down-regulation of p-p70S6K compared with single agents, and this may explain the enhanced growth inhibitory effects of the drug combination. Interestingly, the MEK/ERK cascade has been demonstrated to control cell motility in HCC cell models, through a mechanism involving urokinase receptor (uPAR)-mediated phosphorylation/activation of p70S6K (65). Previous studies have shown that both regorafenib, in HCC and gastric cancer (66, 67), as well as palbociclib, in other cancer types (68, 69), can affect cell motility. Here, we demonstrate that palbociclib and regorafenib combination reduced migration and invasion more efficaciously than individual treatments, possibly through inhibition of p70S6K activity.

It is worth noting that a contribution to p70S6K inhibition might come from AMPK, which was activated by both palbociclib and regorafenib, confirming previous observations (51, 55, 70). The AMPK/mTORC1/p70S6K axis may play a role in the enhanced cell toxicity promoted by the combined treatment of palbociclib with regorafenib. Indeed, a variety of studies demonstrated that activation of AMPK in HCC cells may lead to apoptotic cell death through different mechanisms, including the induction of endoplasmic reticulum stress or autophagy (71). In addition, an AMPK/mTOR-dependent mechanism has been involved in the induction of apoptosis in leukemia cells by suppressing *c*-myc expression (72). Of note, regorafenib abrogated palbociclib-induced expression of cyclin D1, possibly through MAPK and p70S6K signaling inhibition (33), and this might contribute to cell death, as suggested by the previous observation that silencing cyclin D1 promotes apoptosis in HCC cells (73).

Mechanistically, activation of AMPK by palbociclib has been attributed to the inhibition of protein phosphatase 5 (PP5) (55). On the other hand, regorafenib-mediated activation of AMPK in HCC cells was presumably consequence of the energy stress caused by the perturbation of mitochondrial function, as previously demonstrated for sorafenib in breast cancer cells (24). Indeed, regorafenib treatment reduced the basal and ATP-linked respiration as well as the maximal and spare respiratory capacities, in accordance with previous studies (43, 51). In contrast, palbociclib was ineffective, and the impairment of mitochondrial respiration observed upon exposure to palbociclib and regorafenib combination may be attributed only to the action of regorafenib.

The mTORC1/p70S6K signaling is a known critical regulator of energy metabolism (74) and we previously demonstrated that its inhibition, associated with a persistent activation of AMPK, hinders glucose utilization in sorafenib-treated breast cancer cells (24). Also, the CDK4/6-Rb-E2F pathway plays a key role in the control of both cell cycle progression and energy metabolism, through multiple mechanisms that include the involvement of the E2F target *c*-myc (42). A variety of evidence suggest that the metabolic outcomes associated with CDK4/6 inhibition are context specific and CDK4/6 inhibitors may have different and even opposing effects on energy metabolism in different cancers or in the same cancer type depending on the conditions (15).

Here, we show that palbociclib and regorafenib alone reduced or had no effect on glucose uptake depending on the cell line and on the oxygen availability (normoxia or hypoxia), even though both down-regulated mTORC1/p70S6K signaling and *c*-myc, and attenuated the hypoxia-induced expression of HIF-1α and GLUT-1 in all cell models. This suggests that other mechanisms, possibly involving different glucose transporters, may be activated in some conditions by HCC cells to ensure their glucose supply.

Palbociclib and regorafenib combination promoted a down-regulation of glucose uptake in HepG2 and HUH7 cells, although without potentiating the effects of single agent treatments, presumably because their inhibitory action converges on the same regulatory pathways.

Accordingly, the drug combination reduced the expression of HK2, PFKP, and MCT4 in HepG2 cells, and significantly decreased the ECAR under both normoxia and hypoxia/CoCl_2_ treatment, even though no further enhancements were promoted in comparison with single treatments. The relevance of targeting these genes finds support in previous studies demonstrating their key role in HCC tumor growth (44, 75, 76).

Interestingly, the expression of aldolase A was not affected by palbociclib or regorafenib alone, but was significantly reduced by their combination. This glycolytic enzyme belongs to moonlighting enzymes, exerting functions independent of its catalytic activity, such as control of cytokinesis and cell motility through interaction with actin cytoskeleton (77, 78), and transcriptional regulation of genes engaged in the G1/S cell cycle transition (79). It is conceivable that inhibition of such functions may contribute to the anti-tumor efficacy of palbociclib/regorafenib combination.

PKM2 is another moonlighting glycolytic enzyme that exerts nuclear-related pro-proliferative functions when maintained in the less active dimeric form through phosphorylation at Tyr^105^ (47). Palbociclib and regorafenib combination reduced phospho-PKM2^Tyr105^ levels. Although, we did not provide a direct demonstration for the inhibition of PKM2 nuclear translocation, this event might play a role in the anti-tumor activity of the drug combination, as suggested by previous findings showing that sorafenib-mediated dephosphorylation of PKM2^Tyr105^ is critical for the sensitivity to this drug in HCC cells (80).

As a final consideration, the down-regulation of HIF-2α mediated by regorafenib and palbociclib combination may be critical for sustaining the inhibition of glucose metabolism under hypoxia. Indeed, HIF-2α, which preferentially controls the expression of genes not directly related with glycolysis, such as VEGF, Oct4, and cyclin D1, can also regulate enzymes in the glycolytic pathway when HIF-1α is inhibited (50). Therefore, palbociclib and regorafenib combination, by reducing both HIF-1α and HIF-2α expression, might counteract the metabolic adaptation that would favor cell survival under hypoxic conditions.

## Conclusions

Even though palbociclib and regorafenib do not act in synergism to target energy metabolism, our study demonstrates that their combination in HCC cells enhances cell cytotoxicity and inhibits migration and invasion more efficaciously than individual treatments, thereby suggesting a potential strategy for improving the anti-tumor efficacy of regorafenib.

## Data Availability Statement

The raw data supporting the conclusions of this article will be made available by the authors, without undue reservation.

## Author Contributions

Conception and design: CF, GD, MB, and PGP. Cell biology and molecular biology experiments: GD, CF, MB, SLM, and DC. Statistical analysis: AC. Writing of the manuscript: CF and GD. Review of the manuscript: MG, RA, and PB. Study supervision: PGP and GM. All the authors contributed to revise the manuscript and approved the final version for publication.

## Conflict of Interest

The authors declare that the research was conducted in the absence of any commercial or financial relationships that could be construed as a potential conflict of interest.
