# Supplementary material for: Simultaneous Combination of the CDK4/6 Inhibitor Palbociclib With Regorafenib Induces Enhanced Anti-tumor Effects in Hepatocarcinoma Cell Lines
Source: Front Oncol. 2020 Sep 23;10:563249. doi: 10.3389/fonc.2020.563249 (PMC7539564; doi:10.3389/fonc.2020.563249)
Supplement: Supplementary file 1 [file Data_Sheet_1.docx]

Supplementary Material

A B

**Figure S1.** (A) HepG2 cells were treated with 0.1 μM P or with increasing concentrations of Sorafenib (S) (from 1 μM to 10 μM) alone or in simultaneous combination. After 72h, the cells were counted using the trypan blue dye exclusion method. Data are expressed as percent versus control cells. ***p<0.001 vs C; ^###^p<0.001 vs P; ^§§^p<0.01, ^§§§^p<0.001 vs S. (B) HepG2 cells were treated with 0.1 μM P or with increasing concentrations of R (from 1 μM to 10 μM) alone or in simultaneous combination. After 72h, the cells were counted using the trypan blue dye exclusion method. Data are expressed as percent versus control cells. ***p<0.001 vs C; ^###^p<0.001 vs P; ^§§^p<0.01, ^§§§^p<0.001 vs R. All the data are mean values ±SD of two independent experiments.
